# A Review Pertaining to SARS-CoV-2 and Autoimmune Diseases: What Is the Connection?

**DOI:** 10.3390/life12111918

**Published:** 2022-11-18

**Authors:** Nina Kocivnik, Tomaz Velnar

**Affiliations:** 1 Faculty of Pharmacy, University of Ljubljana, 1000 Ljubljana, Slovenia; 2 Department of Neurosurgery, University Medical Centre Ljubljana, 1000 Ljubljana, Slovenia

**Keywords:** COVID-19, SARS-CoV-2, autoimmune diseases, immune response, inflammation

## Abstract

Coronavirus disease 2019 (COVID-19) is an infectious viral disease caused by Severe Acute Respiratory Syndrome Coronavirus 2 (SARS-CoV-2). It is known that infection with SARS-CoV-2 can lead to various autoimmune and autoinflammatory diseases. There are few reports in the literature on the association between SARS-CoV-2 and autoimmune diseases, and the number of reports has been increasing since 2020. Autoimmune diseases and SARS-CoV-2 infections are intertwined in several ways. Both conditions lead to immune-mediated tissue damage, the immune response is accompanied by the increased secretion of inflammatory cytokines and both conditions can be treated using immunomodulatory drugs. Patients with certain autoimmune diseases, such as systemic lupus erythematosus, rheumatoid arthritis, type 1 diabetes, cardiac sarcoidosis, idiopathic pulmonary fibrosis, autoimmune hepatitis, multiple sclerosis and others, are more susceptible to SARS-CoV-2 infection, either because of the active autoimmune disease or because of the medications used to treat it. Conversely, SARS-CoV-2 infection can also cause certain autoimmune diseases. In this paper, we describe the development of autoimmune diseases after COVID-19 and the recovery from COVID-19 in people with autoimmune diseases.

## 1. Introduction

The 2019 coronavirus disease pandemic (COVID-19) has swept across the globe since December 2019 and has become a major public health problem [1,2]. Although the number of studies on the association between autoimmune diseases and severe acute respiratory syndrome coronavirus 2 (SARS-CoV-2) infection has been increasing recently, the issue of the association between autoimmune diseases and SARS-CoV-2 infection has not yet been thoroughly investigated. There are few reports in the literature on how autoimmune diseases can facilitate SARS-CoV-2 infection and worsen the clinical picture of COVID-19 and how the virus can trigger autoimmune diseases in people who have recovered from SARS-CoV-2 infection. In this article, we present the main similarities in the immune responses of the two conditions, the development of autoimmune diseases after COVID-19 and the recovery from COVID-19 in people with autoimmune diseases.

SARS-CoV-2 is a novel coronavirus (CoV) that causes pandemic respiratory infectious disease COVID-19 with a fatality rate of approximately 2% [3,4,5]. The disease first emerged in December 2019 in Wuhan, China, and was officially declared a pandemic by the World Health Organisation (WHO) on 11 March 2020. The number of deaths due to COVID-19 is estimated at more than 6.4 million [6,7,8]. SARS-CoV-2 is a positive-polar single-stranded RNA (ssRNA) virus belonging to the genus Betacoronavirus group 2B. Genomic analyses have shown that it has 79.6% genomic sequence identity to SARS-CoV, which caused the outbreak of severe acute respiratory syndrome (SARS) in 2002, and 50% sequence homology to MERS-CoV, which caused the Middle East respiratory syndrome (MERS) epidemic in 2012–2013 [5,9,10]. The virus has four structural proteins: a spike protein (protein S), an envelope protein (E), a membrane protein (M) and a nucleocapsid protein (N). Protein S, with its receptor binding domain (RBD), plays a key role in infection. The target of the virus has been identified as the lung epithelium. The receptor binding domain (RBD), located in the S1 subunit of protein S, allows viral entry into host cells via the angiotensin-converting enzyme 2 (ACE2) receptor [5,6]. The S1/S2 polybasic cleavage site, which is proteolytically cleaved by the cellular cathepsin L and the transmembrane protease serine 2 (TMPRSS2) protease, is also important for infection [9]. The latter facilitates virus entry at the plasma membrane surface, whereas the lysosomal cysteine protease cathepsin L facilitates virus entry into cells lacking TMPRSS2 by activating protein S in endosomes [9,11,12]. SARS-CoV-2 is transmitted directly by respiratory droplets, as evidenced by the productive replication of SARS-CoV-2 in the upper and lower respiratory tract and by the many cases of human-to-human transmission of the virus during close contact with active coughing [9]. The virus passes through the nasal and laryngeal mucosa and, after binding to airway epithelial cells, enters the alveolar epithelial cells in the lung. From there, it can enter the peripheral blood and infect cells of tissues expressing the ACE2 protein, such as the heart, kidneys, gastrointestinal tract and brain [10,13]. The usual clinical manifestation of the virus is fever, fatigue, dry cough, headache, dyspnoea and sore throat. Olfactory and taste disturbances may also occur. Highly pathogenic CoV causes severe influenza-like symptoms that can progress to acute respiratory distress, pneumonia, renal failure or even death [6,9,14]. In most people, signs of infection appear after an incubation period of 1 to 14 days (most commonly around 5 days), and dyspnoea and pneumonia develop within eight days of onset of illness. All age groups are susceptible to SARS-CoV-2 infection, but the clinical picture varies according to age. Those aged 60 years and over are more likely to develop severe respiratory disease resulting in hospitalisation or, in severe cases, death, while most young people have mild or even asymptomatic symptoms [10]. The WHO reports that 80% of infections are mild to moderate and 13.1% develop severe disease. In 6.1% of those infected, critically severe disease requiring intensive treatment may develop [5]. Risk factors for the development of a more severe course include age over 60 years, high blood pressure, obesity, cardiovascular disease, diabetes mellitus, pregnancy, underlying lung disease and relative immunodeficiency [1,5]. Some people may develop long-lasting effects of infection, known as long COVID, after infection with SARS-CoV-2. This is defined by signs and symptoms that develop during or after infection that are consistent with COVID-19, last more than 12 weeks and cannot be explained by an alternative diagnosis. They are usually manifested by clusters of symptoms that often overlap, can fluctuate and change over time and can affect any system in the body. Although long COVID often develops in people who have had a severe form of COVID-19, the condition can develop in anyone who has been infected with SARS-CoV-2 and especially in those who are being treated for various autoimmune diseases [15,16].

The immune system protects the body from foreign bodies, viruses, microbes and from infected or tumour cells. To function normally, the immune system must be able to distinguish between foreign bodies and the body’s own antigens [17]. Autoimmunity is an immune response against the body’s own antigens. Autoimmune disease occurs when tolerance to self-antigens breaks down. Antibodies that attack the body’s own cells are called autoantibodies and the body consequently starts to produce antibodies against its own cells. The immune system works in the same way in autoimmune diseases as it does in defence against foreign bodies. Autoimmune diseases are caused by a disturbance in immune regulation. The disruption is caused by changes in the interaction between B and T lymphocytes and antigen-presenting cells (APCs). Antibodies that bind to self-antigens are produced. This results in immune complexes that activate complement or T lymphocytes that mature into cytotoxic lymphocytes, macrophages or natural killer (NK) cells. As a consequence, lytic mechanisms are triggered that are characteristic of complement activation or cell-mediated immune responses. This leads to tissue damage and inflammation [18,19,20]. Autoimmune diseases are influenced by both one’s own genes and the environment. Genes can directly affect the cells of the immune system by altering autoreactivity. Changes in tissue function are manifested as altered lymphocyte and immune function. Environmental factors include the products of microorganisms (e.g., bacterial DNA and viruses). These factors can enhance the immune response to antigens and thus stimulate the natural immune system, thereby preventing the destruction of foreign bodies [17,21]. There are two broad groups of autoimmune diseases: organ-specific and systemic autoimmune diseases. In organ-specific autoimmune diseases, individual organs are affected, whereas in systemic autoimmune diseases, it is mainly organ-nonspecific antigens that are attacked (consequently, multiple organs are affected). These are either found in the bloodstream (e.g., rheumatoid factors) or are components of cells (e.g., anti-DNA antibodies) [18]. Each disease is characterised by unique antibodies that detect and target the antigens. Some of these antigens are located on a single organ, causing an organ-specific autoimmune disease, while others are located on multiple organs, causing a systemic autoimmune disease. Some of the more common organ-specific autoimmune diseases are as follows: autoimmune thyroid disease, type 1 diabetes, psoriasis, multiple sclerosis and Guillain–Barré syndrome. Common systemic autoimmune diseases include systemic lupus erythematosus, rheumatoid arthritis, chronic inflammatory bowel disease, Sjögren’s syndrome and antiphospholipid syndrome [22,23].

## 2. Connections between COVID-19 and Autoimmune Diseases

There are several links between COVID-19 and autoimmune diseases. In both diseases, chronic inflammation with immune-mediated tissue damage is at the core [6,24]. In autoimmune diseases, tissue damage occurs due to sustained inflammatory reactions resulting from loss of immune tolerance due to immune dysfunction. This leads to damage and malfunction of various organs. As SARS-CoV-2 infection triggers immune reactions in the host, such immune-mediated damage also occurs in severe COVID-19 [20,25].

The excessive release of inflammatory cytokines and chemokines is a feature of both SARS-CoV-2 infection and autoimmune diseases, both of which can cause severe damage to various organs. Increased levels of interleukin-1 (IL-1), interleukin-2 (IL-2), interleukin-6 (IL-6), interleukin-8 (IL-8), interleukin-10 (IL-10), interleukin-17 (IL-17), interleukin-18 (IL-18), CXC chemokine ligand 10 (CXCL10) and CXC chemokine ligand 2 (CCL2) have been detected in SARS-CoV-2 infections. The level of expression of certain cytokines and the degree of cytokine imbalance influence the severity and outcome of the disease. Similar to autoimmune diseases, damage-associated molecular patterns (DAMPs), i.e., molecules released from damaged or dead cells as a result of infection or injury, can also worsen the course of infection and disease outcome [6,7,24,25].

In patients with COVID-19, the researchers confirmed extrafollicular B-cell activation and neutrophilia, as well as excessive macrophage activation resulting from cytokine storm. This is also characteristic of people with autoimmune diseases. This inflammatory state leads to the production of excessive inflammatory cytokines, the polarisation of activated macrophages towards an M1 inflammatory phenotype and cytotoxic dysfunction, which can ultimately lead to complications and a severe outcome of COVID-19. The activation of neutrophil granulocytes and the production of neutrophil extracellular traps (NETosis) play an important pathogenic role in COVID-19. Increased serum levels of neutrophil extracellular traps are also present in some autoimmune diseases, such as antiphospholipid syndrome [6,7,24,26]. Another important similarity between COVID-19 and autoimmune diseases is the use of immunomodulatory drugs and biologic agents targeting inflammatory cytokines. Thus, corticosteroids, Janus kinase (JAK) inhibitors, IL-1 blockers and IL-6 receptor antagonists have been used to treat some severe forms of COVID-19 [6,7,14,24,27].

The following sections describe the similarities in pathogenesis between COVID-19 and the most common and perhaps best-studied autoimmune diseases to date, such as systemic lupus erythematosus, rheumatoid arthritis and multiple sclerosis.

### 2.1. Systemic Lupus Erythematosus

Systemic lupus erythematosus (SLE) is a chronic systemic autoimmune disease in which the immune system attacks and damages its own tissues and organs, most commonly the joints, skin, lungs, kidneys and central nervous system [28]. In SLE patients, the cytokine interferon gamma (IFN-γ) plays a key role in the pathogenesis of SLE, while IL-23 and IL-17 cause kidney problems [29,30]. Experience to date shows that patients with COVID-19 also have kidney problems, which is due to an increase in inflammatory cytokines in SARS-CoV-2 infection [31,32]. People with SLE have been shown to have increased levels of follicular helper T cells 2 and 17 (cTfh2 and cTfh17), which leads to the increased secretion of interleukin-21 (IL-21). This promotes the induction of inflammatory states by stimulating and proliferating B cells and the consequent production of autoantibodies that damage tissues and organs. Another common point of similarity in the pathogenesis of COVID-19 and SLE is interleukin-22 (IL-22), which plays a key role in the regulation of antiapoptotic proteins, influencing serum amyloid A (SAA) levels and fibrinogen production. SAA is increased in serum due to activation of the signal transducer and activator of transcription 3 (STAT3) on the IL-22 receptor on the surface of hepatocytes or due to induction of the expression of various cytokines. SLE patients have elevated serum SAA levels, and a study has shown that people with COVID-19 also have higher than average serum SAA levels [33]. Both COVID-19 and SLE patients also have changes in serum fibrinogen levels [29,30].

### 2.2. Rheumatoid Arthritis

Rheumatoid arthritis (RA) is a chronic systemic autoimmune disease that damages the lining of the joints and causes swelling. The inflammation that occurs in rheumatoid arthritis can damage other parts of the body, such as the skin, eyes, lungs, heart and blood vessels, in addition to the joints, and these patients are at increased risk of viral infections, including SARS-CoV-2 [34]. In inflammatory diseases, angiotensin II stimulates the production of prostaglandins and vascular endothelial growth factor (VEGF), which accelerates the inflammatory response, increases vascular endothelial permeability and the accumulation of inflammatory cells in damaged tissues [29]. Angiotensin II also increases TNF-α and IL-6 gene expression in endothelial cells, macrophages and fibroblasts and increases C-reactive protein (CRP) levels. Patients with COVID-19 and RA have increased levels of angiotensin II, which, by decreasing the expression of angiotensin-converting enzyme 2 (ACE2), also leads to reduced vascular permeability and the possibility of lung injury. Elevated angiotensin II levels and a decrease in ACE2 increase the risk of further lung injury, which may occur in acute respiratory distress syndrome (ARDS). Thus, the clinical use of ACE2 inhibitors may increase the severity of disease states in patients with COVID-19 and RA. This means that the use of ACE2 inhibitors in patients with COVID-19 is not beneficial as it increases the effect of angiotensin II. Conversely, reducing ACE levels in RA patients has beneficial effects as it reduces the effects of angiotensin II [29,35,36].

### 2.3. Multiple Sclerosis

Multiple sclerosis (MS) is an organ-specific autoimmune disease that results from damage to the myelin of the nerve fibres of the central nervous system, which can eventually lead to neurological damage [37]. The pathogenesis of MS is not yet fully understood. It is known that lymphocytes migrate across the blood–brain barrier and cause inflammation in the central nervous system. Elevated levels of inflammatory cytokines, such as IFN-γ, IL-12, tumour necrosis factor-alpha (TNF-α) and IL-17, are detected in people with MS. IL-6 and transforming growth factor-beta (TGF-β) stimulate increased secretion of IL-17 and IL-21 and thus the differentiation of Th17 T helper cells via the activation of STAT3, which is characteristic of various autoimmune diseases, including MS. IL-17, in addition to osteopontin, plays an important role in the pathogenesis of MS. Osteopontin prevents the programmed cell death of T cells, macrophages, endothelial cells and fibroblasts, which contributes to the initiation of inflammation and damage to the myelin sheath. As in MS patients, inflammatory cytokine levels are increased in COVID-19 patients. An increase in Th17, which regulates inflammatory states by producing IL-6 and IL-23, has been detected in SARS-CoV-2-infected humans. This state plays a key role in the development of cytokine storm, which can lead to ARDS and multi-organ failure [7,29,38,39,40].

## 3. Development of Autoimmune Diseases after SARS-CoV-2 Infection

People may experience a number of symptoms in the post-COVID-19 period, the most common of which are exhaustion, difficulty concentrating, headaches, loss of smell or taste, chest, joint or muscle pain [41]. In addition to these complications, studies have also reported new-onset autoimmune and autoinflammatory diseases resulting from SARS-CoV-2 infection [6,7,42,43,44,45]. Three mechanisms explain the development of autoimmune diseases in these patients: molecular mimicry, epitope spreading and bystander activation [7,42]. Other causes of autoimmune disease following this infection include long-standing viral infections with other viruses and a hyperinflammatory innate immune response that increases autoimmune responses and thus susceptibility to autoimmune disease [44].

Molecular mimicry is one of the key mechanisms involved in the formation of autoantibodies and thus in the development of autoimmune diseases. Antigenic molecular mimicry can occur as a result of exposure of the immune system to viral antigen epitopes that are structurally almost identical to human protein sequences. This provokes the cross-reactivity of the antibody. This means that specific antibodies or effector T cells against the microbial antigens are able to react with the corresponding host antigens, resulting in autoimmune disease. A classic example of molecular mimicry in autoimmune diseases is the immune response to Epstein–Barr virus in lupus patients [6,24,44]. Cross-reactivity with autoantigens has also been observed in SARS-CoV-2. SARS-CoV-2 contains structural protein S, which is responsible for viral entry into the cell. The pentapeptide sequence of this protein corresponds to the sequence of 24 human surfactant-like peptides, 13 of which are located on SARS-CoV-2 immunoreactive epitopes. The S protein heptapeptides, however, mimic 26 peptides of the human proteome, some of which encode human proteins responsible for the clinical complications of COVID-19 (7).

Epitope spreading is defined as the ability of the body to respond to cryptic (masked) epitopes one by one by providing antigen-presenting cells to self-reactive lymphocyte clones with cryptic epitopes of their own antigens [44]. The presentation of cryptic antigens may be the result of a viral infection that causes tissue damage, thereby unmasking previously hidden antigens. The exposed antigens may become the target of an immune response, and the resulting inflammatory response only enhances the unmasking of these antigens. The antigens are then presented to autoreactive T lymphocytes, triggering an immune response [45].

**Figure 1:**
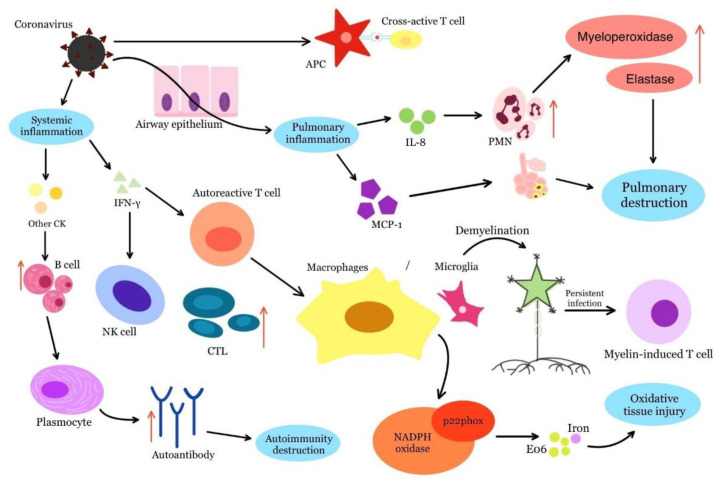
Several molecular mechanisms may link coronavirus infection and autoimmunity: (I) the coronavirus infection starts local pulmonary inflammation. Hyper-innate inflammatory response leads to the accumulation of alveolar macrophages, the stimulation of polymorphonuclear cells with proteolytic enzyme secretion (elastases and myeloperoxidases) and increased MCP-1 and IL-8 production, finally leading to pulmonary damage; (II) molecular mimicry may active cross-reactive T-cells because viral antigens mimic host antigens; (III) the production of inflammatory cytokines trigger B-cells and consequent autoimmune tissue destruction by autoantibodies. IFN-γ is crucial in these pathways, as it activates autoreactive T-cells, natural killer cells and stimulates cytotoxic lymphocytes, resulting in the oxidative tissue injury by macrophages and demyelination by microglia in the central nervous system (IL-8, interleukin-8; CK, cytokines; CTL, cytotoxic lymphocyte; MCP-1, monocyte chemotactic protein 1; IFN-γ, interferon-γ; red arrow indicates an increase).

The activation of neighbouring antigenically non-specific immune cells occurs during a virus-induced immune response. The release of cytokines activates autoreactive T and B cells in an antigen-independent manner, triggering autoimmunity. The resulting inflammatory environment causes tissue damage and exposure to non-specific antigens, which stimulates immune cell activation [7]. Such a process has been described in murine hepatitis virus infection (JHM strain) and serves as an animal model for the development of autoimmune diseases, for example in MS. In this disease, according to this animal model, demyelination is triggered by the activation of T cells after infection with the JHM virus. This process is dependent on IFN-γ, which in turn can activate autoreactive T cells at the site of infection. These cells then move to the site of chronic inflammation and interact with microglia or macrophages to induce the activation of adjacent antigen-nonspecific immune cells (Figure 1) [44].

Persistent infection with other coronaviruses is another possible mechanism for the development of autoimmune diseases after SARS-CoV-2 infection. Antiviral antibodies may be involved in the development of autoimmune diseases, and a persistent T cell reaction against virus-infected cells results in chronic inflammation. This is the case in JHM infection, where both SR T cells and myelin-specific CD4+ T cells contribute to persistent viral infection. In the chronic infection phase, the severity of demyelination is consistent with a tendency for myelin-induced T cells rather than SR T cells to activate adjacent antigen-nonspecific immune cells [44].

Viral infections alter leukocyte counts in the host, impair liver enzyme function and increase plasma inflammatory cytokines and chemokines. The increase in plasma inflammatory cytokines and chemokines is referred to as a hyperinflammatory innate response. The hyperinflammatory innate response causes an accumulation of alveolar macrophages and stimulates polymorphonuclear neutrophils to secrete myeloperoxidases and elastases, accompanied by an increase in monocyte chemoattractant protein-1 (MCP-1) and IL-8. In this way, the hyperinflammatory innate response upsets the immune balance and causes lung injury [29,44].

### 3.1. Autoimmune Diseases after COVID-19

Infection with SARS-CoV-2 can upset the immune balance and trigger autoimmune responses, thereby triggering the development of various autoimmune diseases [6]. In a study of 987 patients hospitalised for SARS-CoV-2 pneumonia, autoantibodies to type I interferon (IFN-I) were detected in 101 patients, and 40 patients had autoantibodies to all 13 types of interferon alpha (IFN-α). Thus, an association between SARS-CoV-2 infection and the presence of autoantibodies associated with antigens present in certain tissues has been demonstrated, which may explain the development of autoimmune and inflammatory multisystem diseases [45]. Other studies have also demonstrated the presence of autoantibodies in patients with severe COVID-19 [46,47]. These autoantibodies are most commonly antinuclear and antiphospholipid antibodies, which are also present in autoimmune diseases [48] (Table 1). The most frequently reported autoimmune diseases that occurred after COVID-19 include Guillain–Barré syndrome (GBS), autoimmune haemolytic anaemia, antiphospholipid syndrome, systemic lupus erythematosus, multiple sclerosis, acute disseminated encephalomyelitis, multisystem inflammatory syndrome in children (MIS-C) and rheumatoid arthritis [5,6,7,15,42,43,44,45]. Here, we describe the most common and currently best-studied autoimmune diseases occurring in healthy people after COVID-19, such as Guillain–Barré syndrome, antiphospholipid syndrome and Kawasaki disease in the context of MIS-C in children.

#### 3.1.1. Guillain–Barré Syndrome

Guillain–Barré syndrome (GBS) is an organ-specific autoimmune disease in which autoantibodies are directed against gangliosides, a component of the myelin sheath that envelops axons in peripheral nerves [49]. This results in impaired stimulus conduction, causing muscle paralysis and sometimes sensory disturbances. Some studies describe the onset of neurological symptoms 7 to 10 days after the onset of respiratory symptoms characteristic of COVID-19 [50,51,52]. Although SARS-CoV-2 antibodies cannot be detected in cerebrospinal fluid in most cases of GBS, Gigli et al. reported a case of GBS with a positive test for SARS-CoV-2 antibodies in cerebrospinal fluid in a study they conducted. The results of this study was able to provide evidence that SARS-CoV-2 infection can cause GBS, but the possible mechanisms of this disease and its association with COVID-19 are still unknown [6]. An increased number of vascular events, such as venous and arterial thrombosis and stroke, have also been described in patients with COVID-19 who developed GBS. These are associated with the presence of anti-phospholipid autoantibodies, elevated levels of which are associated with increased neutrophil release, increased platelet levels and renal problems. High levels of these autoantibodies are associated with increased release of neutrophil granulocytes and neutrophil extracellular traps (NETs), increased platelet levels, renal and pulmonary impairment. Serum blood samples from patients who died of COVID-19 were analysed and found to have elevated levels of autoantibodies against annexin A2, a protein required for the maintenance of the pulmonary microvasculature. SARS-CoV-2 infection triggers an autoimmune response similar to GBS. The autoantibodies that are produced are directed against annexin A2, leading to blood vessel damage, mainly in the lungs. Incidentally, a similar mechanism of tissue damage has been described in autoimmune haemolytic anaemia and thrombocytopenic purpura, suggesting that COVID-19 is a complex disease that also has serious chronic consequences associated with lung, kidney and cardiovascular damage [24,43,45].

#### 3.1.2. Antiphospholipid Syndrome

Antiphospholipid syndrome (APS) is a systemic autoimmune disease in which arterial and venous thrombosis occurs. Serologically, APS is defined by the persistent presence of antiphospholipid antibodies (aPL), which have also been detected at high levels in patients with COVID-19 [53,54,55]. This could be evidence that SARS-CoV-2 infection triggers APS. aPL may also occur transiently in various other infections. Coagulopathy and thrombotic events leading to pulmonary embolism and stroke are sometimes present in patients with severe COVID-19. In these patients, D-dimer is the most important abnormality in coagulation parameters and its increasing value is used as a prognostic parameter for a worse outcome. The role of aPL in thrombotic complications in SARS-CoV-2 infected patients is not yet clear. As with COVID-19, thromboses in multiple organs can occur in a short time in APS. The similarity in the course of COVID-19 and APS has led to speculation that SARS-CoV-2 infection might trigger the development of APS. Therefore, various studies have been conducted on the presence of aPL in COVID-19. In this study, however, aPL testing was not repeated after infection, so it is not known whether the presence of antibodies was only transient or permanent [56]. In another study, aPL testing was repeated, but here aPL was not clearly associated with thrombotic complications [57]. Another study confirmed the presence of aPL in COVID-19 but found low aPL titres in contrast to APS, which is characterised by high aPL titres [58]. Thus, the studies are conflicting and suggest that the presence of aPL in COVID-19 may be only transient, and in particular, there is no strong evidence that aPL may play a role in the increased coagulation propensity in the setting of COVID-19 in patients with APS [7,45,58].

Clinically, CRP has an important value in APS. CRP is an acute inflammatory protein, which values increase during infections, damage of tissue and also during autoimmune diseases. Very high values of CRP are documented in bacterial infections. In viral respiratory infections, the CRP values are lower. Higher levels of CRP during COVID-19 infection have been used as an indicator of disease severity, as they indicate a severe infection course [59,60]. One significant feature of CRP in clinical practice is that CRP has a known affinity for phospholipids. Activated partial thromboplastin time (aPTT) is one of several blood coagulation tests. It was proven that CRP may interfere with aPTT, prolonging the clotting time in proportion to the CRP concentration. Thus, the elevated CRP prolongs the aPTT in samples, regardless of whether an anticoagulant is added or not. Serum aPL have also been linked to COVID-19 and autoimmunity. These antibodies are a sign of APS and may be encountered in 1% to 5% of the healthy population and up to 18% of adults with chronic diseases. The serum aPL titres may increase with age [56,61]. Raised CRP levels, as an indicator of inflammation, are also observed in patients with APS and correlate with the severity of the clinical picture [56,60,61,62]. Since only the presence of aPL is not enough to develop the APS, a second trigger is needed, which is usually an infection. For APS diagnosis is characteristic persistence of high titres of lupus anticoagulant, anticardiolipin antibodies IgG and IgM or anti-β2glycoprotein-1 IgG and IgM for more than 12 weeks [63]. High levels of lupus anticoagulants have also been documented in patients with COVID-19. It is not known, however, whether antiphospholipid antibodies were newly produced with COVID-19 or were increased or previously present in serum [64,65]. According to some studies, aPL were present in 47% of critically ill patients with COVID-19. These antibodies have been detected five to six weeks after the COVID-19 infection, indicating that a long-lasting course of the disease increases the risk of developing APS [61,62].

#### 3.1.3. Kawasaki Disease in MIS-C

Multisystem inflammatory syndrome in children (MIS-C) is a systemic disease occurring in the paediatric population and includes Kawasaki disease, Kawasaki disease shock syndrome, toxic shock syndrome, myocarditis and macrophage activation syndrome. Since the onset of the COVID-19 pandemic, there has been a sudden increase in the number of patients with MIS-C, and scientists have begun to investigate why and how MIS-C develops in association with SARS-CoV-2 infection [7,42,43,45]. As mentioned previously, certain diseases can occur as a consequence of infection with, for example, Epstein–Barr virus, cytomegalovirus or other coronaviruses. The exact mechanism by which SARS-CoV-2 infection causes MIS-C is not yet fully understood. One hypothesis is that this pathological mechanism is related to events that develop after infection, rather than to the infection itself. Late events include the antibody production and blockage of IFN I and III by the virus, which may lead to late cytokine storm and consequent MIS-C. Another possible mechanism is autoantibody formation by molecular mimicry and vascular injury due to deposition of immune complexes [45]. MIS-C is clinically manifested by fever, gastrointestinal and cardiovascular manifestations and elevated levels of inflammatory cytokines in SARS-CoV-2 infection. MIS-C is very similar to Kawasaki disease, which is an acute systemic vasculitis caused by an excessive immune response to infection in children. Kawasaki disease (KD) is a systemic autoimmune disease whose symptoms include the inflammation of the medium-sized arteries, most commonly the coronary arteries. MIS-C and KD are very similar but the main difference is that MIS-C is more common in children older than 5 years [7]. During the duration of the COVID-19 pandemic, there have been various cases of an increase in the incidence of KD [43]. For example, a research group in Italy described a 30-fold increase in the incidence of KD, and eight out of ten patients with KD-like illness were positive for IgG or IgM to SARS-CoV-2 [66]. On May 12th 2020, the European Centre for Disease Prevention and Control (CDC) reported a total of 224 cases of MIS-C in Europe [67]. Eight children with features of hyperinflammatory shock similar to atypical Kawasaki disease, Kawasaki disease shock syndrome or toxic shock syndrome were reported from London. All were positive for SARS-CoV-2 antibodies [68]. From these reports, it is possible to see that MIS-C symptoms associated with COVID-19 overlap with KD but symptoms not usually associated with KD are also present [43]. Because many patients have an incomplete form of KD during the COVID-19 infection, it is crucial to distinguish between KD and COVID-19-associated Kawasaki disease (KD-COVID-19) (Table 2). Compared to classical KD, the patients with KD-COVID-19 are older and can have gastrointestinal and meningeal signs, leukopenia with marked lymphopenia, thrombocytopenia, increased ferritin or signs of myocarditis. KD-COVID-19 is also associated with the increased incidence of myocarditis and cardiac involvement and can therefore result in more frequent hospitalisation in intensive care [68,69]. Additional symptoms include frequent shock and hemodynamic failure, meningeal and gastrointestinal symptoms and laboratory abnormalities, such as significant lymphopenia and leukopenia, thrombocytopenia, high procalcitonin, ferritin, cardiac enzymes and troponins [62,66]. There have been specific changes in the coronary arteries, observed in 25% patents. These have been documented on coronary angiograms, including asymptomatic dilatation or aneurysms. In some severe cases, larger aneurysmal dilatations of coronary arteries with resultant thrombosis and myocardial infarction were documented [62,66,69,70]. Therefore, the patients with KD-COVID-19 have a more austere disease course than those with classic KD. It is, however, important to note that the evidence of SARS-CoV-2 infection is needed to confirm the diagnosis of KD-COVID-19. For effective treatment, organ failure prevention and lowering mortality, the early diagnosis and treatment of COVID-19 are of vital importance. Treatment included intravenous immunoglobulin administration supported by steroid therapy [62,71,72].

Some genetic diseases and congenital anomalies are a risk factor for developing severe COVID-19 and the genetic abnormalities have also been linked to autoimmune disorders [73,74]. The studies in patients with SARS-CoV-2 have discovered some uncommon innate immunity errors in the interferon pathways and in the immune signalling circuits [74]. Besides the elevated levels of the inflammatory cytokines that have been connected to a higher mortality in COVID-19 patients, the aberrant activation of the immune system and hyperinflammation may also predispose the individuals to autoimmune diseases. One of such most notable hemophagocytic lymphohistiocytosis (HLH) and multisystem inflammatory syndrome (MIS) [73,74,75].

HLH is a rare condition, which is fatal in a high percentage of patients. It is characterised by systematic inflammatory disorder and dysfunction of multiple organs, which has also a genetic origin [73,75]. Besides acquired risk factors in non-inherited form, also an inherited form of the disease exists, based on the genetic defects. HLH is characterised by an aberrant activation of the immune system, including a highly stimulated but ineffective immune activation of inflammation cells and resultant cytokine storm. Central cells include the T cells and macrophage/monocytes, which are continuously activated and accumulate in various organs, including spleen, liver and bone marrow. Due to a release of cytokines in high concentrations, hyperinflammation ensues, resulting in organ damage. This is also an aggravating factor for worsening of the SARS-CoV-2 infection [75,76,77].

MIS is a hyperinflammatory condition due to abnormal immune response and numerous organ system involvement that occurs after SARS-CoV-2 infection, affecting children and adults [78]. It has been discovered that inborn errors of immunity at eight genetic loci govern TLR3-dependent IFN induction, amplification or response to IFN, implicating defects in TLR3, TRIF, TBK1, IRF3, UNC93B, IRF7 and IFNAR1/2 [79]. Although rare, these patients with inborn errors of immunity showed that type I IFN immunity is essential for the SARS-CoV-2 infection and that defects in these circuits predispose individuals to critical COVID-19 related worsening of the infection [74].

The acute inflammation in critical COVID-19 infection has major similarities to inflammatory conditions, such as macrophage activation syndrome and haemophagocytic lymphohistiocytosis, whereas MIS has a high resemblance to toxic shock syndrome and Kawasaki disease, the latter also affecting children and being characterized by fever, inflammation and autoimmune damage to endothelium, heart and gastrointestinal tract [80,81,82]. The HLH and Kawasaki disease share a genetic basis and can be activated by viral infection. The genes that are responsible for autoinflammation, immune dysregulation and autoimmunity may cause a gain of function or loss of inhibition of pathways responsible for of cytokine and TLR signalling cascade regulation, especially those linked to IL1 and IL6 [82,83]. In MIS, the genetic defects may be partly related to tissue type, given that SARS-CoV-2 antigen-mediated TCR Vb21.3 polyclonal T cell expansion and activation seem to play a major role in the pathogenesis. The genetic components linking MIS and COVOD-19 are still incompletely understood [74,84].

As a therapeutic option, immunosuppression has been suggested and preliminary results are encouraging [85,86]. A therapy including corticosteroids, immunoglobulins and immunoadsorption in order to mitigate inflammation may help to alleviate the consequences of cytokine storm and concomitant organ damage [73,86].

## 4. COVID-19 in Patients with Autoimmune Diseases

Since the outbreak of the COVID-19 pandemic, many questions have been raised about the risk of SARS-CoV-2 infection and associated complications in people with autoimmune diseases [14,87]. It is still unclear whether or not autoimmune diseases pose a high risk of severe complications [2,6,7,14,15]. Results from some studies suggest a higher rate of hospitalisation and more complications of COVID-19 in people with autoimmune diseases and a higher mortality rate [6,14,15,24,88,89,90,91]. People with autoimmune diseases could be more susceptible to SARS-CoV-2 infection compared with the healthy population due to impaired immune response, use of immunosuppressive drugs and damage to various organs [6,15,29]. Other sources report a higher susceptibility to infection in patients with autoimmune diseases, either because of active autoimmune disease or because of the drugs used to treat them [7]. On the other hand, the results of other studies do not show an association between more severe COVID-19 trajectories in people with autoimmune diseases [6,7,87,88,90,92]. However, the results of some studies have suggested that people with autoimmune diseases may have a protective factor that would protect them from the most severe complications of COVID-19 as a result of taking medication [2,7,90].

Many people with autoimmune diseases who are receiving immunosuppressive drugs have stopped treatment for fear of the side effects of these drugs, which may increase susceptibility to SARS-CoV-2 infection, leading to faster viral replication, more severe symptoms and a more severe disease course [6,7,14,88]. For example, the use of non-steroidal anti-inflammatory drugs (NSAIDs) is associated with higher complication rates in SARS-CoV-2 respiratory tract infections [7,14]. Although there is an association between the use of NSAIDs and adverse respiratory effects, there is still no unequivocal evidence to support the need for urgent discontinuation of treatment in the setting of SARS-CoV-2 infection [7,93]. Corticosteroids, such as dexamethasone, shorten the duration of mechanical ventilation and reduce mortality in people with severe SARS [15,94]. However, the US Center for Disease Control and Prevention has advised against corticosteroid therapy because of the potential prolongation of viral replication [2]. Conventional synthetic disease-modifying antirheumatic drugs (csDMARDs) are widely used for the treatment of autoimmune rheumatic diseases. Among them, methotrexate is widely used for the treatment of RA. According to the literature, methotrexate increases the number of herpes zoster viral infections, and the results of a meta-analysis performed on RA patients confirmed an increased risk of all adverse respiratory effects [95]. However, these results are limited to non-COVID-19 studies. For reliable data, a study of SARS-CoV-2-infected patients treated with methotrexate would be needed [14,88]. The use of TNF-α inhibitors (etanercept, infliximab, adalimumab) is also thought to be effective in reducing the risk of developing a more severe course of COVID-19 [14]. On the other hand, it is known that individuals treated with TNF-α inhibitors are more susceptible to infections and have a more severe course of disease [1,7]. Steroid use is also thought to increase the risk of COVID-19 infection due to immune suppression [1,88]. The antirheumatic drug tocilizumab, which is used for the treatment of RA, is thought to effectively improve the clinical symptoms of COVID-19 [14,87]. Janus kinase inhibitors (JAK inhibitors), such as filgotinib, ruxolitinib, baricitinib and upadacitinib, which are used in the treatment of psoriasis, rheumatoid arthritis, myelofibrosis and chronic inflammatory bowel disease, act by inhibiting tyrosine kinase, which is involved in viral transmission, to reduce viral infectivity and thereby prevent more severe inflammation. They also inhibit the intracellular transmission of various inflammatory cytokines such as IFN-I and IFN-γ, thus preventing the primary antiviral response. Therefore, the use of Janus kinase inhibitors has been proposed for the treatment of more severe forms of COVID-19 [7,14,88]. Glucocorticoids, as immunosuppressive drugs, increase susceptibility to SARS-CoV-2 infection, slow the clearance of the virus from the body and ultimately lead to a higher risk of a severe course of the disease [7,91].

Some drugs for systemic autoimmune rheumatoid diseases that act directly or indirectly on cytokines involved in cytokine storm could be used to treat COVID-19 [7]. Chloroquine and hydroxychloroquine are antimalarial drugs used for the treatment of various autoimmune rheumatic diseases such as SLE and RA. Both drugs increase endosomal pH, leading to a decrease in IFN-I levels, thereby negatively affecting viral binding to the receptor and strongly inhibiting its replication. Antimalarial administration also alters the glycosylation of the ACE2 receptor and affects the transport of SARS-CoV-2 from endosomes to endolysosomes [2,7,14]. Because of the potential efficacy of chloroquine and hydroxychloroquine in the treatment of SARS-CoV-2 infections, a number of studies are underway, with varying results [88,89,90,91,92,93,94,95,96,97,98,99,100,101,102]. In some studies, the drugs did not prevent COVID-19 or reduce hospitalisation rates in people with autoimmune rheumatic diseases [88,89,90,91,92,93,94,95,96,97,98,99,100,101]. On the other hand, another study reported a reduction in viral transmission in 20 people with COVID-19 who received hydroxychloroquine [101]. The described autoimmune diseases, their medical treatment and possible connections with COVID-19 are summarised in Table 3.

The CDC has published a list of high-risk physiological and medical conditions, i.e., those at higher risk of serious illness from SARS-CoV-2. Among these, some autoimmune diseases have been described, such as autoimmune myocarditis, Dressler syndrome, subacute bacterial endocarditis, cardiac sarcoidosis, idiopathic pulmonary fibrosis, autoimmune hepatitis, multiple sclerosis, Guillain–Barré syndrome, myasthenia gravis, lupus, rheumatoid arthritis, scleroderma and Sjögren’s syndrome [15]. Thus, current data suggest that people with certain autoimmune diseases have an increased risk of SARS-CoV-2 infection and possible complications, whereas other autoimmune diseases do not. People with thyroid problems, such as Hashimoto’s disease, are not at an increased risk of developing COVID-19, according to current data. Inflammatory bowel disease (IBD), which includes Crohn’s disease and ulcerative colitis, is treated with immunosuppressive drugs. Therefore, people with IBD are likely to be more susceptible to SARS-CoV-2 infections. The susceptibility to SARS-CoV-2 infection in people with coeliac disease is currently reported to be the same as in the general population [88].

### 4.1. Systemic Lupus Erythematosus

People with SLE are at increased risk of many types of infections, including colds, influenza and infection with other viruses, with SARS-CoV-2 being no exception [88]. It is also one of the leading causes of death in SLE patients, with approximately 30% of deaths related to infections, the most common of which are respiratory infections. SLE patients with higher levels of IL-17, IFN-γ and IL-23 are more likely to develop severe clinical conditions. Th17, as a key factor in the pathogenesis of SLE, is not only a source of IFN-γ secretion but also causes renal impairment by stimulating the synthesis of IL-23 and IL-17. ACE2 is a receptor for SARS-CoV-2 on the surface of endothelial cells and is also expressed in a number of organs, notably the kidney and lung. Viral particles present in endothelial cells can induce apoptosis due to the accumulation of inflammatory cells in this area. One of the critical factors in SLE patients is the malfunctioning of the apoptosis mechanism. A number of factors associated with the pathogenesis of SLE, such as infections and toxins, can cause increased apoptosis of infected cells. During apoptosis, nuclear antigens become accessible to the immune system and lead to the increased production of antibodies and cytokines. These cytokines cause cell death by accumulating in the cell. This makes SLE patients with higher ACE2 levels more susceptible to SARS-CoV-2 infection [29,30,103].

Fedratinib is a JAK2 inhibitor. Its effect on patients with COVID-19 is good because it reduces the risk of cytokine storms. For the same reason, anifrolumab, which is an antibody against the IFN-α receptor, has a good impact. Chloroquine and hydroxychloroquine also have a good effect on the recovery from COVID-19 by interfering with ACE2 glycolysis and reducing cytokine storm formation. Belimumab is an antibody against the soluble stimulator of lymphocyte B. It prevents the production of inflammatory cytokines from B cells and thus has a good effect on COVID-19 patients by reducing the intensity of the immune response [29,104].

### 4.2. Rheumatoid Arthritis

Based on the currently known data, it is not yet clear whether individuals with RA have an increased risk of SARS-CoV-2 infection [88]. Scientists have not yet identified the cause of the immune dysfunction in RA but genetic factors; environmental factors, such as viral and bacterial infections; and hormonal factors play an important role in causing the impaired immune response. ACE2 converts angiotensin II to angiotensin-1–7. Angiotensin II uses Janus kinase to trigger activity via the Ras/Raf/MAPK signalling pathway. Under inflammatory conditions, angiotensin II stimulates the production of prostaglandins and vascular endothelial growth factor, thereby triggering inflammatory responses and increasing vascular permeability. Genetic factors play an important role in increasing the risk of COVID-19 in RA patients. The polymorphisms of the ACE2 gene may play a role in the worsening of the condition of patients infected with the virus. In general, polymorphisms in the ACE2 gene may be present in COVID-19 patients with severe clinical conditions. Therefore, ACE2 gene polymorphisms can be considered as a prognostic factor. RA patients are also at an increased risk of SARS-CoV-2 infection due to the use of immunosuppressive drugs, which increase the risk of developing viral infections [29,35,36].

Tocilizumab and sarilumab are antibodies against the IL-6 receptor that may have a good effect on the course of COVID-19 by inhibiting IL-6 binding to its receptors and reducing the activity of inflammatory cytokines. Quinapril and ramipril also have a good effect by preventing the production of angiotensin II and its associated inflammatory effects [29].

### 4.3. Multiple Sclerosis

As mentioned in the above paragraphs, the pathogenesis of MS remains unclear. Due to the lack of data on SARS-CoV-2 infection in MS patients, it is not yet known whether people with MS have a higher risk of SARS-CoV-2 infection than healthy people [29]. Corticosteroids used for the relief of MS suppress the immune system and thus lead to increased susceptibility to infection. Conversely, however, it is theorised that immune suppression may also play a protective role against SARS-CoV-2 infection; in fact, a weakened immune system may prevent the production of inflammatory cytokines and inhibit the occurrence of respiratory complications in these patients [29,38,86,88].

Drugs used to treat multiple sclerosis have different effects on the course of COVID-19. Alemtuzumab has a poor effect due to the removal of T cells, which are a defensive barrier against the virus. Ocrelizumab and rituximab have a good effect by reducing the amount of interleukins produced by B cells. Tocilizumab has a good effect by reducing the production of cytokine storms. Etanercept also has the same effect. Natalizumab also has a good effect by limiting leukocyte binding and trafficking [29].

### 4.4. Type 1 Diabetes Mellitus

Diabetes mellitus is a condition of high blood glucose levels. There are several types of diabetes but the most common are type 1 (insulin-dependent type) and type 2 (non-insulin-dependent type). Type 1 diabetes is autoimmune-mediated, where it is an organ-specific autoimmune disease [22,105]. The pathogenesis of diabetes is complex, as many factors play a role in its development. For example, the production of antibodies against pancreatic islet cells causes these cells to function abnormally and increases blood sugar levels. On the other hand, abnormalities in organs such as the liver, adipose tissue and skeletal muscle are also responsible for the development of diabetes. Impaired insulin secretion reduced the sensitivity of target tissues and increased glucose production in the liver are three metabolic abnormalities in diabetes. Dietary fat activates signalling through TLR2 and TLR4 receptors and triggers endoplasmic reticulum stress. Signalling via TLRs increases the secretion of inflammatory cytokines by inducing the transcription of inflammatory genes. On the other hand, TLR2 and TLR4 increase the activity of intracellular tyrosine kinase, which inhibits insulin signalling through this mechanism. Activated inflammatory mediators interfere with the production of insulin from pancreatic islet cells, as these cells are damaged by the damaging action of inflammatory cells that invade the islets. Eventually, this process leads to insufficient insulin secretion and thus to hyperglycaemia in people with diabetes. In this disease, neutrophil and Th1 cell function, chemotaxis, phagocytosis and intracellular killing are impaired and impaired immune responses are also observed. These abnormalities make diabetic patients more vulnerable to infections, especially respiratory infections, and serious complications [29,106]. Uncontrolled diabetes is particularly important as hyperglycaemia can impair immune function [62]. In a study conducted in Wuhan, 42.3% of the deceased SARS-CoV-2-infected patients were diabetic [107].

The risk of diabetes and SARS-CoV-2 infection is higher in overweight patients and people who consume high-fat diets because of ongoing inflammatory activity, inadequate immune activation and the involvement of macrophages and TNF-α in these conditions. Modulating blood sugar levels in overweight people may be a way to reduce their risk of developing diabetes and SARS-CoV-2 infection [29].

## 5. Conclusions

The link between SARS-CoV-2 and autoimmune diseases appears undisputed but several long-term studies are required to help us understand the effect. In some respects, the immune response of the two conditions is very similar, ranging from the excessive release of inflammatory cytokines to B cell activation and excessive macrophage activation, which can lead to damage to various organs. The pathogenesis of COVID-19 and more common autoimmune diseases, such as systemic lupus erythematosus, rheumatoid arthritis and multiple sclerosis is also quite similar. Mechanisms, such as molecular mimicry, epitope spreading, the activation of neighbouring antigenically non-specific immune cells and others, explain the possible occurrence of autoimmune diseases in healthy people after COVID-19. The most frequently reported autoimmune diseases following SARS-CoV-2 infection include Guillain–Barré syndrome, antiphospholipid syndrome and Kawasaki disease in the context of multisystem inflammatory syndrome in children. People with pre-existing autoimmune diseases stopped immunomodulatory treatment during the COVID-19 pandemic, fearing an increased risk of SARS-CoV-2 infection. Questions have also been raised about the increased risk of a severe course of COVID-19 and the higher mortality rate in patients with autoimmune diseases. Thus, due to the conflicting results of previous studies, we cannot conclude with certainty whether people with autoimmune diseases are at higher risk compared to the healthy population. A particular aspect is the use of drugs for the treatment of rheumatic diseases, such as chloroquine and hydroxychloroquine, which could be used for the treatment of COVID-19 due to their effect on cytokines. In the future, it will be necessary to gather more research results on SARS-CoV-2 to better understand the link between autoimmune diseases and SARS-CoV-2 infections.

## Figures and Tables

**Table 1:** Autoantibodies and their clinical significance.

Autoantibody	Clinical Significance
Anti-IFN-I antibody	Causes severe forms of COVID-19
Anti-IFN-α antibody	Possible emergence of autoimmune diseases and worsening of active COVID-19 infection
ANA	Poor prognosis
aPL	Poor prognosis
Anti-A2Ab	Worsens pulmonary symptoms during COVID-19 infection

**Table 2:** The main differences between the classic KD and KD-COVID-19.

	Classic KD	KD-COVID-19
Age	patients younger than 5 years	older patients (~7.5 years)
Gastrointestinal and meningeal manifestations	atypical	usually present
Blood count	leukocytosis, mild normocytic anemia, thrombocytosis, elevated erythrocyte sedimentation rate or CRP	leukopenia with marked lymphopenia, thrombocytopenia
Procalcitonin, ferritin, cardiac enzymes, troponin	elevated	even higher
Signs of myocarditis	may occur	increased incidence
Coronary angiograms	coronary artery aneurysms may lead to myocardial ischemia	larger aneurysmal dilatations of coronary arteries with resultant thrombosis and myocardial infarction
Steroid therapy	may be used	is commonly used
Effect of IVIG on CAAs	good response	resistance to therapy is common

**Table 3:** Autoimmune diseases, their medical treatment and possible connections with COVID-19.

Autoimmune Diseases	Medications	Drug Function	Response in Autoimmune Patients	Possible Response in COVID-19 Patients
Systemic lupus erythematosus	Fedratinib	JAK2 inhibitor	Good	Good (could possibly reduce the risk of cytokine storm)
	Anifrolumab	Antibody against the IFN-α receptor	Good	Good (could possibly reduce the risk of cytokine storm)
	Chloroquine and hydroxychloroquine	Antimalarial drugs that have negative effect on viral binding to the receptor and its replication	Good	Good (interferes with ACE2 glycolysis and reduces cytokine storm formation)
	Belimumab	Antibody against soluble stimulator of lymphocyte B	Good	Good (reduces the intensity of the immune response)
Rheumatoid arthritis	Tocilizumab and sarilumab	Antibodies against the IL-6 receptor	Good	Good (inhibits the IL-6 binding to its receptors and reduces the cytokines inflammatory activity)
	Quinapril and ramipril	ACE inhibitors	Good	Good (prevents the angiotensin II production and its associated inflammatory effects)
Multiple sclerosis	Alemtuzumab	Anti-CD52	Good	Not good (removes T cells, which help prevent viral infections)
	Ocrelizumab and rituximab	Anti-CD20	Good	Good (reduces the amount of interleukins)
	Tocilizumab	IL-6 receptor blocker	Good	Good (could possibly reduce the risk of cytokine storm)
	Etanercept	Anti-TNFα	Not good	Good (could possibly reduce the risk of cytokine storm)
	Natalizumab	Antibody against CD49d	Good	Good (limits leukocyte binding and trafficking)
